# Two-dimensional crystal engineering using halogen and hydrogen bonds: towards structural landscapes†
†Electronic supplementary information (ESI) available. See DOI: 10.1039/c7sc00129k



**DOI:** 10.1039/c7sc00129k

**Published:** 2017-03-16

**Authors:** Arijit Mukherjee, Joan Teyssandier, Gunther Hennrich, Steven De Feyter, Kunal S. Mali

**Affiliations:** a Division of Molecular Imaging and Photonics , Department of Chemistry , KU Leuven-University of Leuven , Celestijnenlaan 200F , B3001 Leuven , Belgium . Email: steven.defeyter@kuleuven.be ; Email: kunal.mali@kuleuven.be; b Universidad Autonoma de Madrid , Cantoblanco , 28049 Madrid , Spain

## Abstract

Two-dimensional (2D) crystallization on solid surfaces is governed by a subtle balance of supramolecular and interfacial interactions. However, these subtle interactions often make the prediction of supramolecular structure from the molecular structure impossible. As a consequence, surface-based 2D crystallization has often been studied on a case-by-case basis, which hinders the identification of structure-determining relationships between different self-assembling systems. Here we begin the discussion on such structure-determining relationships by comparing the 2D crystallization of two identical building blocks based on a 1,3,5-tris(pyridine-4-ylethynyl)benzene unit at the solution–solid interface. The concepts of supramolecular synthons and structural landscapes are introduced in the context of 2D crystallization on surfaces to identify common structural elements. The systems are characterized using scanning tunneling microscopy (STM). This strategy involves carrying out minor structural modifications on the parent compound to access supramolecular patterns that are otherwise not obtained. We demonstrate that this chemical perturbation strategy translates equally well for 2D co-crystallization experiments with halogen bond donors yielding porous bi-component networks. The holistic approach described here represents a stepping stone towards gaining predictive power over the 2D crystallization of molecules on solid surfaces.

## Abstract 2

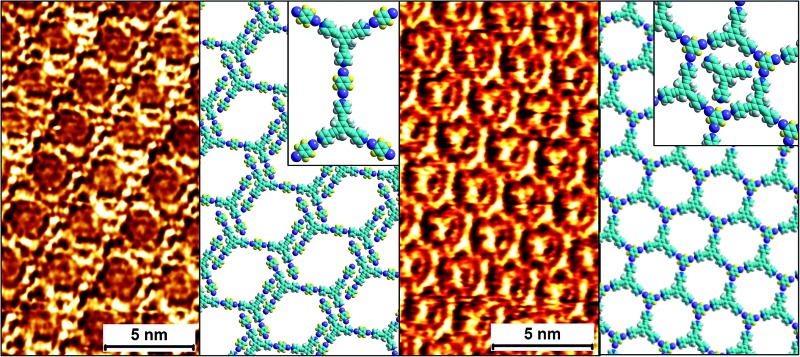
We apply the concepts of supramolecular synthons and structural landscapes to 2D crystallization at the solution–solid interface.

## Introduction

Molecular self-assembly on solid surfaces, although known for years, has been widely studied over the past few decades using scanning tunneling microscopy (STM)^1–3^ and low-energy electron diffraction (LEED).^4^ The self-assembly of planar organic molecules on solid surfaces typically leads to the formation of crystalline monolayers. Such monolayers can be formed at the solution–solid interface^5^ or under ultrahigh vacuum (UHV) conditions at the UHV–solid interface.^6^ In both instances, the monolayers are stabilized through supramolecular interactions between the adsorbed molecules and interfacial interactions that prevail between the molecules and the solid surface. As well as a fundamental interest in understanding molecular organization on surfaces, such physisorbed self-assembled monolayers are intensively investigated due to their importance in bottom-up nanofabrication methods.^7,8^

The formation of supramolecular networks on surfaces, especially those obtained at the solution–solid interface, is frequently likened to bulk crystallization, albeit with reduced dimensionality. Surface confinement significantly reduces the number of possible ways in which molecules can pack. Two-dimensional (2D) crystallization thus presents a relatively simplified scenario where only 17 plane groups are sufficient to describe the possible symmetry element combinations, in comparison to the 230 space groups needed to describe the packing in 3D. Despite this simplification, 2D crystallization on surfaces remains relatively complex due to competitive intermolecular and interfacial interactions. Unlike bulk crystal structures, which are mainly governed by intermolecular interactions and the principle of close-packing, 2D crystallization is usually strongly dependent on the nature of the underlying surface. As a consequence, the outcome of the process is often different from that in the bulk. Only in cases where the intermolecular interactions overwhelmingly dominate the network formation, the molecule–surface interactions have negligible influence on 2D self-assembly leading to predictable surface patterns that may resemble the bulk crystal structure. Such surface patterns are often incommensurate with the surface lattice. Furthermore, molecules are free to adapt suitable conformations in 3D. In 2D on the other hand, they tend to adsorb in a planar conformation on solid surfaces to maximize molecule–surface interactions. Last but not the least, self-assembly in 2D often features a concentration-dependent component where the structure depends on the concentration of the solution. This phenomenon of concentration-dependent structure formation (*vide infra*) is absent in bulk crystallization. While one can design systems where the 2D and 3D crystal structures are analogous,^9–11^ the scope of such an approach is often limited and may not be practical when predictive power over 2D crystallization is sought.

Unlike crystal engineering in bulk, which has greatly benefited from the Cambridge Structural Database (CSD),^12^ which allows researchers from across the discipline to access statistical information on crystal packing, research on 2D crystallization has been obscured by the lack of a systematic compilation of structural data. Such information allows the identification of structure-determining relationships that are otherwise difficult to discern from isolated examples. A notable exception is the 2D structural database (2DSD).^13^

Structural polymorphism is one of the widely investigated facets of 2D crystallization where the self-assembly of a single building block leads to multiple different crystalline networks. A number of factors influence the formation of one structure over another. A non-comprehensive list of such factors includes the solvent,^14,15^ solution concentration,^16–18^ substrate,^19^ temperature^20,21^ and thermal history of the sample.^22,23^ Concentration-dependent network formation is a routinely observed phenomenon in 2D crystallizations. Certain molecules form densely packed networks at higher concentrations whereas dilute solutions yield low-density or so called ‘nanoporous’ networks.^24^ Self-assembled network formation is typically governed by the maximization of molecule–molecule and molecule–substrate interaction energies scaled per unit area. This condition, however, changes as the solution concentration is lowered. Network formation from dilute solutions is dominated by molecule–molecule interaction energies (not scaled per unit area) thus producing patterns dissimilar to those observed at higher concentrations. Concentration-controlled structure formation is a fundamental factor in understanding molecular assembly on solid surfaces.^25^

In order to be technologically relevant, the outcome of 2D crystallization processes should be predictable.^26^ The research on bulk crystallization also faced the same conundrum a few decades ago, which eventually led to the concept of supramolecular synthons. Supramolecular synthons are structural units identifiable within crystals, which can be obtained *via* known synthetic operations involving intermolecular interactions.^27^ These are kinetically defined units which depict the spatial arrangement of intermolecular interactions thereby providing an approximation of how the whole crystal would look. The synthon approach assists in the prediction of crystal structures from molecular structures. This concept, which was introduced with the dual objective of reducing the complexity and enhancing the predictability of supramolecular synthesis, is being increasingly employed in the rational design and synthesis of novel crystals with desired properties.^28,29^

Another emerging concept in crystal engineering is that of structural landscapes.^30^ Although different routes are possible, crystallization often proceeds *via* discrete nucleation pathways. The existence of polymorphs often indicates the presence of such energy-related nucleation events which correspond to specific crystallization routes. Recent studies have shown that minor chemical modifications on the parent compound allow the sampling of different possible polymorphic structures. The collection of all such observed structures represents a map of packing possibilities for related systems and thus constitutes the crystal structure landscape.^31^ The concept of structural landscapes has strengthened the notion that crystal structures, when looked at from a holistic viewpoint, give insights into the crystallization mechanism itself.^32^

The two concepts described above allow for the identification of similarities and differences between crystal structures within a family of compounds. In 2D crystallizations however, such correlation is non-existent. As mentioned earlier, the self-assembled networks for a given molecular system are not unique and depend on a number of experimental variables. A convergent approach, which allows for the identification of supramolecular synthons within structurally related families of compounds, will be valuable for achieving predictability in 2D crystallizations. The comparison of the self-assembled networks of structurally related compounds will provide the basis of generalizations *vis-à-vis* supramolecular synthons. Specifically, monitoring the structural changes observed in self-assembled networks in response to minor chemical modifications on the parent building block is a promising approach.

**Fig. 1:**
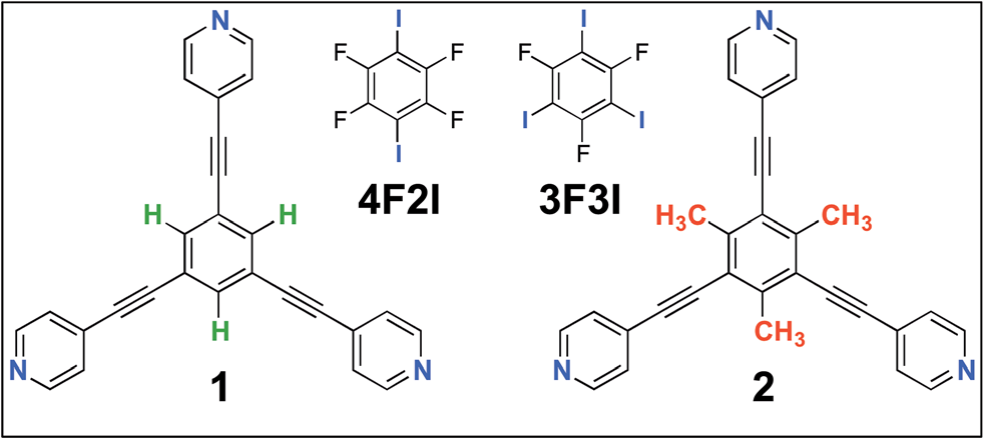
The molecular structures of the compounds. The 2D crystallization of **1** ^35^ and its co-crystallization with halogen-bond donors such as **4F2I** and **3F3I** ^34^ has been reported earlier. The present study reports on the 2D crystallization of **2** and its co-crystal formation with **4F2I** and **3F3I**.

In this contribution, we present a rational strategy for accessing an average 2D structural landscape of a class of compounds based on a 1,3,5-tris(pyridine-4-ylethynyl)benzene unit (**1**, Fig. 1). We show that when there are many kinetic possibilities in the crystallization process that lie within a narrow energy window, but only a few are accessible in the form of crystal structures, rational chemical perturbation of the parent system can provide access to alternative 2D crystallization pathways. This concept is illustrated using a structurally similar derivative bearing a mesityl core^33^ instead of phenyl (**2**, Fig. 1). We also demonstrate that this structural perturbation strategy translates equally well for co-crystals, allowing their formation under equilibrium conditions, which was otherwise considered impossible. The work presented here reveals that simple molecular substitution eases the challenges associated with the co-crystallization of **1** with halogen bond donor molecules such as **4F2I** and **3F3I** (Fig. 1).^34^ While we do not compare the crystallization behavior of these molecules in 3D with that observed in 2D, we apply the concepts of supramolecular synthons and structural landscapes from bulk crystal engineering to understand the 2D crystallization of structurally related derivatives of 1,3,5-tris(pyridine-4-ylethynyl)benzene.

## Results and discussion

**Fig. 2:**
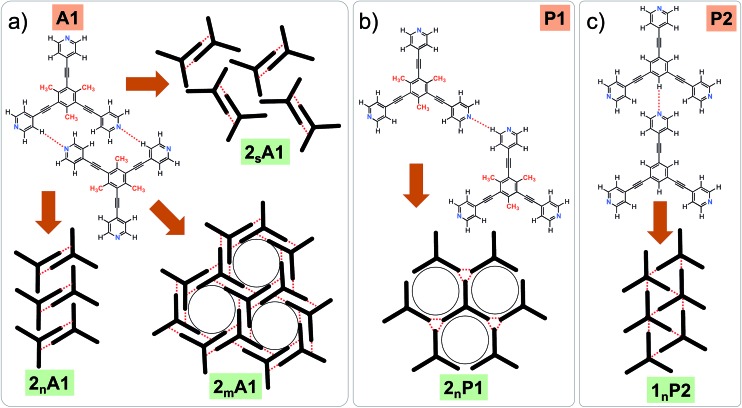
(a–c) Primary synthons based on N(pyridyl)···H–C(pyridyl/aryl) interactions (red dotted lines). These three primary synthons give rise to three different structures. **P** stands for the parallel arrangement of dimers whereas **A** stands for the antiparallel arrangement. While all three synthons, namely **A1**, **P1** and **P2**, can be formed in the case of compound **1**, only two possibilities exist for compound **2**, as primary synthon **P2** cannot be formed due to a lack of hydrogen bonding interactions. Panel (a) shows all the experimentally observed structures for compound **2**. Panel (b) shows a hypothetical low-density network (not observed experimentally) for compound **2**. Panel (c) shows the close-packed network based on synthon **P2** for compound **1**. This network is stabilized by N(pyridyl)···H–C(pyridyl) hydrogen bonds. m, n and s are arbitrary letters used to identify the different structures.

**Fig. 3:**
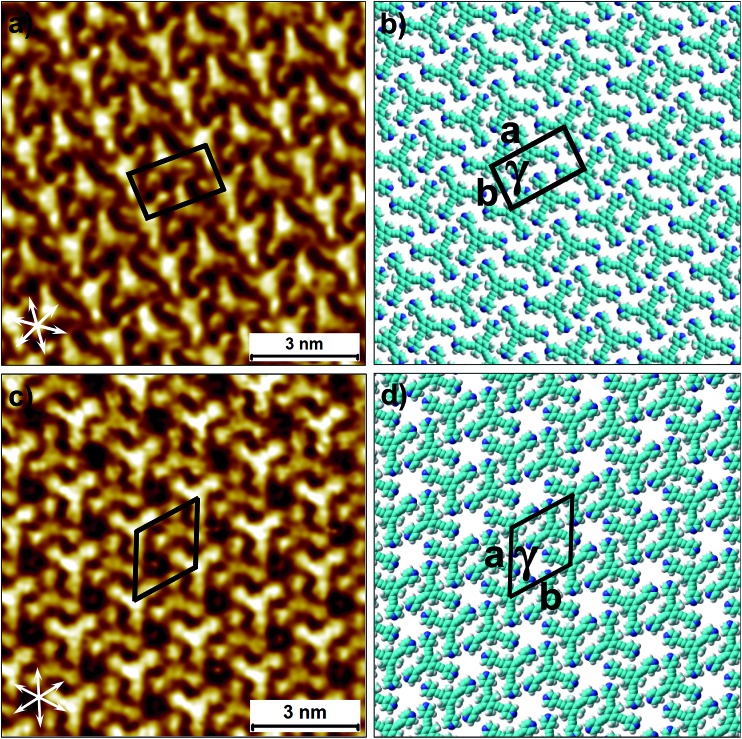
Concentration-dependent 2D crystallization of **2** at the 1-phenyloctane/HOPG interface. (a) STM image of the **2_n_A1** network (*C* = 250 μM). The arrows in the lower left corner represent the graphite symmetry axes. (b) Molecular model depicting the arrangement of molecules in the **2_n_A1** network. (c) STM image of the **2_m_A1** network (*C* = 25 μM). (d) Molecular model depicting the arrangement of molecules in the **2_m_A1** network. Imaging parameters: *V*_bias_ = –500 mV and *I*_t_ = 100 pA. The unit cell parameters are provided in Table 1.

Fig. 1 shows the molecular structures of **1** and **2**. The two molecules differ only slightly. The hydrogen atoms on the central phenyl ring in **1** (green) are replaced with methyl groups in **2** (red). The 2D crystallization of parent compound **1** has been intensively studied in the recent past, both at the solution–solid interface^35^ and under ultrahigh vacuum (UHV) conditions.^36,37^ Based on the molecular geometry and anticipated N(pyridyl)···H–C(pyridyl-aryl) intermolecular hydrogen bonding, three structurally distinct patterns namely **P1**, **P2** and **A1**, hereafter termed as primary synthons, can be obtained (Fig. 2, also see Fig. S1 in the ESI†). The letters P and A refer to the parallel and antiparallel positioning of two molecules within a dimer, respectively. DFT calculations have revealed that the single H-bond energies of the hydrogen-bonded dimers vary as *E* **_P1_** > *E* **_P2_** > *E* **_A1_** for compound **1**.^35^ The STM characterization of the 2D crystallized networks revealed that **1** forms concentration-dependent self-assembled patterns at the 1-phenyloctane/highly oriented pyrolytic graphite (HOPG) interface. At relatively higher concentrations, a high-density supramolecular network (**1_n_P2**) based on primary synthon **P2** was formed, whereas lower concentrations yielded a low-density pattern (**1_n_P1**) based on **P1** (Fig. S2†).^35^ The synthons as well as the extended networks of **1** can be inferred from Fig. 2 (also see Fig. S1 in the ESI†). Given that the hydrogen atoms on the central phenyl ring are replaced by methyl groups in **2**, one readily anticipates that the formation of supramolecular synthon **P2** will be disfavored since the N(pyridyl)···H–C(pyridyl-aryl) intermolecular hydrogen bonds as shown in Fig. 2c cannot be formed in the case of compound **2**. It remains to be seen if supramolecular networks based on primary synthons **P1** and **A1** are formed. To assess the different 2D crystallization possibilities, the concentration-dependent self-assembly of **2** was studied at the 1-phenyloctane/HOPG interface. The dropcasting of relatively concentrated solutions (250 μM) onto the HOPG surface led to the formation of a close-packed pattern. The STM image and corresponding molecular model displayed in Fig. 3a and b, respectively, show that the close-packed network (**2_n_A1**) is based on antiparallel dimer synthon **A1** where the hydrogen-bonded dimers are close-packed in columns. Each molecule of **2** forms four N(pyridyl)···H–C(pyridyl) hydrogen bonds with a neighboring molecule in an **A1** configuration (see Fig. S3 in the ESI†). Although single rows of polymorph **2_n_A1** resemble the **1_n_P2** structure, hydrogen-bonded antiparallel (**A1**) dimers form the basis of the overall structure. It must be noted that for an extended structure with the **P2** synthon, no hydrogen bonds can be formed between neighboring molecules for compound **2** (see Fig. S4 in the ESI†). The preferential formation of the **2_n_A1** network over the hypothetical **2_n_P2** network is thus a clear indication of the stabilization offered by the weak hydrogen-bonding interactions between the neighboring molecules.

**Table 1:** Structural parameters of the different polymorphs of compounds **2** (this work) and **1** (reported).^35^ Additional STM images for the three networks formed by compound **2** are provided in Fig. S7 of the ESI^*a*^

Structure	Primary synthon		Unit cell parameters			*N*/unit cell	Density (N nm^–2^)
			*a* (nm)	*b* (nm)	*γ* (°)		
**2_n_A1**	**A1**	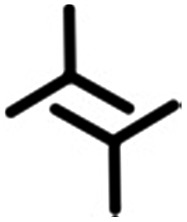	2.3 ± 0.1	1.5 ± 0.1	85.0 ± 1.0	2	0.58
**2_m_A1**	**A1**	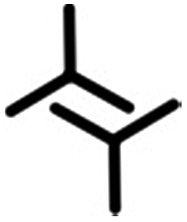	2.1 ± 0.2	2.1 ± 0.2	58.0 ± 4.0	2	0.54
**2_s_A1**	**A1**	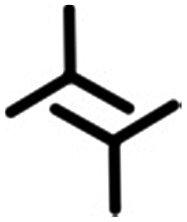	3.4 ± 0.3	2.8 ± 0.1	83.0 ± 2.0	4	0.42
**1_n_P1**	**P1**	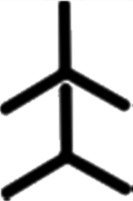	1.33 ± 0.02	1.33 ± 0.02	60.0 ± 2.0	1	0.66
**1_n_P2**	**P2**	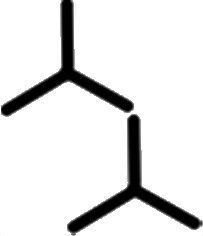	1.62 ± 0.02	1.62 ± 0.02	63.0 ± 2.0	1	0.43

^*a*^ *N* = number of molecules.

**Table 2:** Structural parameters of the 2D co-crystals **2-4F2I** and **2-3F3I** (this work). The structural parameters of **1-4F2I** and **1-3F3I** are also provided for comparison (reported)^34^ ^*a*^

Structure	Unit cell parameters			*N*/unit cell
	*a* (nm)	*b* (nm)	*γ* (°)	
**2-4F2I**	3.3 ± 0.1	3.7 ± 0.1	72.0 ± 3.0	7
**2-3F3I**	2.5 ± 0.1	2.5 ± 0.1	60.0 ± 2.0	3
**1-4F2I**	3.4 ± 0.2	3.6 ± 0.2	79.0 ± 2.0	7
**1-3F3I**	2.5 ± 0.2	2.5 ± 0.2	60.0 ± 2.0	2

^*a*^ *N* = number of molecules.

2D crystallization from dilute 1-phenyloctane solution (25 μM) led to the formation of a relatively low-density pattern. The STM image and molecular model corresponding to the STM data provided in Fig. 3c and d, respectively, show that antiparallel synthon **A1** sufficiently describes this new supramolecular network (**2_m_A1**). The hydrogen-bonded antiparallel dimers are arranged in a cyclic fashion giving rise to a hexagonal porous pattern. Each molecule of **2** forms six N(pyridyl)···H–C(pyridyl) bonds with its neighbors. The supramolecular network exhibits organizational chirality at the level of hexamers and given the absence of any chiral influence on the assembling system, two types of molecular domains, related to each other *via* mirror image symmetry, were observed (see Fig. S5 in the ESI†). The handedness of the hexamers is domain specific meaning that the same chiral motif is preserved throughout a given domain. This low-density network is observed exclusively within the concentration range of 25 μM to 83 μM. At higher concentrations it co-exists with **2_n_A1** with a lower surface coverage. It must be noted that structures based on antiparallel synthon **A1** were not obtained for parent compound **1**. The preferential formation of parallel dimer synthons for **1** occurs due to the higher interaction energies of **P1** and **P2** compared to that of **A1** (*vide supra*).^35^

A comparison of the outcomes of the 2D crystallization of compounds **1** and **2** clearly reveals that simple methyl group substitution strongly influences the preference for the primary synthon of the two compounds. While the supramolecular networks formed by **1** were always based on parallel primary synthons **P1** and **P2**, compound **2** prefers to undergo 2D crystallization *via* selection of the antiparallel primary synthon **A1**. This is also in line with the observation that the introduction of –Me groups often reduces the packing efficiency of the system.^38^ Such a distinct change in the preference of primary synthon as a function of minor changes in the molecular backbone is a characteristic feature of the structural landscapes observed in bulk systems and often indicates that all these structures are part of the same *average* landscape for a given structural class.^39^ Though the absence of synthon **P2** in the 2D crystallization of **2** is readily anticipated due to absence of stabilizing hydrogen-bonding interactions, compound **2**, in principle, is capable of forming supramolecular networks based on synthon **P1**. Based on the known dependence of structure formation on solution concentration^16–18^ and the molecular density of the anticipated low-density network, a further reduction in the solution concentration of **2** may provide a **2_n_P1** network (Fig. 2b). Diluted solutions (<25 μM) however did not yield the **2_n_P1** network. The lack of formation of **2_n_P1** could be related to the lower adsorption energy of **2** compared to that of **1**. The presence of methyl groups on the central phenyl ring is expected to reduce the molecule–substrate contact thereby destabilizing relatively low-density networks such as **2_n_P1**.

The structural landscape strategy allows us to examine the self-assembled networks of **1** from a broader perspective. While compound **1** assembles *via* primary synthons **P1** and **P2** at the 1-phenyloctane/HOPG interface, this preference may change depending on the solid surface on which the 2D crystallization occurs and the specific experimental conditions. This fact is reflected in the 2D crystallization of **1** on an Ag(111) surface under UHV conditions where it forms the **1_n_A1** network based on primary synthon **A1**.^36^ As well as the drastically different nature of the interface, the thermal history of the sample is also critical in governing the outcome of 2D crystallization processes.^22,23^ In studies carried out under UHV conditions, the sample is often annealed at high temperature before being subjected to imaging under cryogenic conditions. The formation of **1_n_A1** on the Ag(111) surface indicates that such a structure is indeed a possibility within the average landscape, however it is possibly unfavorable energetically at the solution/HOPG interface. The results presented above clearly reveal that slight chemical perturbation (as in compound **2**) allows access to such a seemingly high-energy structure.

**Fig. 4:**
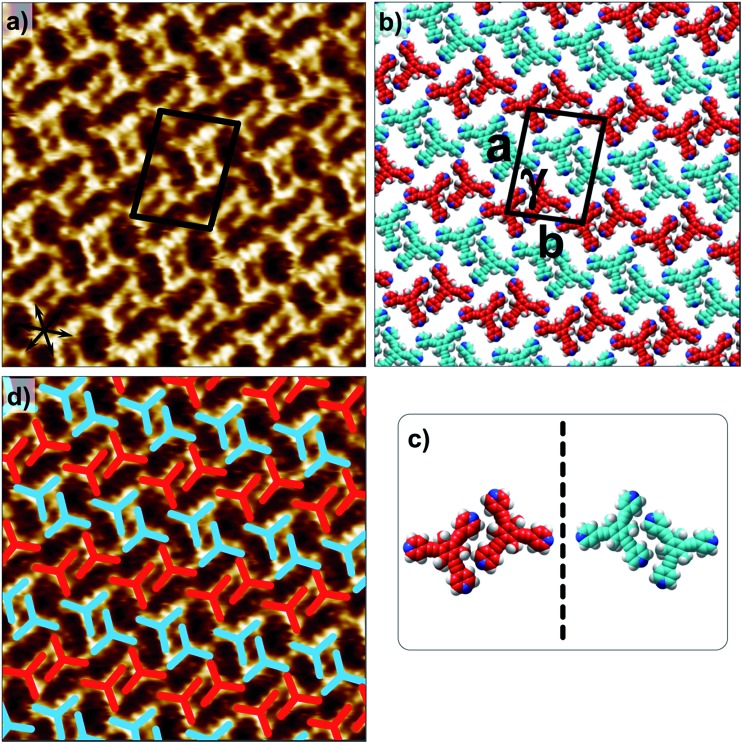
The additional low-density network formed by **2** at the 1-phenyloctane/HOPG interface upon deposition of a dilute solution (25 μM). (a) STM image of the **2_s_A1** network. The arrows in the lower left corner represent the graphite symmetry axes. Imaging parameters: *V*_bias_ = –650 mV and *I*_t_ = 120 pA. (b) Molecular model depicting the arrangement of molecules in the **2_s_A1** network. (c) A schematic diagram showing antiparallel dimer synthons **A1** are related to each other through mirror symmetry. (d) The same STM image as in (a) but overlaid with color-coded dimer synthons related to each other through mirror image symmetry.

As well as the two polymorphs described above, compound **2** forms another pattern upon the deposition of a 25 μM solution onto HOPG. This polymorph (**2_s_A1**, Fig. 4a and b) has the lowest density amongst the networks formed by **2** and is also based on primary synthon **A1**. In contrast to the other two polymorphs, which were consistently observed in the low and high concentration regime, this pattern was observed rather sporadically upon the deposition of dilute solutions. This structure is unique as it is composed of both left-handed and right-handed anti-parallel dimers (Fig. 4c and d) adsorbed within the same domain. The self-assembled monolayer is formed from alternating rows of left-handed and right-handed dimers leading to an overall racemic structure. The exact reason for the formation of this pattern is unclear. The co-adsorption of solvent molecules could possibly explain the lower density of molecules in this pattern. Solvent-induced polymorphism is known to stabilize certain structural patterns in self-assembled monolayers where the solvent plays a more active role and co-adsorbs with the molecules of interest.^14^ In fact, certain 2D polymorphs of 1,3,5-tris(4′-biphenyl-4′′-carbonitrile)benzene (**BCNB**) were found to be stabilized by the co-adsorption of alkanoic acids and 1-phenyloctane, which were used as solvents. It must be noted here that **BCNB** is structurally similar to both **1** and **2**. In the case of **2**, 1-phenyloctane molecules are possibly co-adsorbed in between the adjacent dimers (dark regions in the STM image, also see Fig. S6†) and are not resolved with STM. We note that **BCNB** also forms supramolecular networks based on antiparallel dimer synthon **A1**.^40^ Although **BCNB** is slightly larger in size than **1** as well as **2**, a broadly defined structural landscape allows the inclusion of slightly different molecules within the same landscape.^39,41^ It can be argued that the three molecules are part of the same virtual and average structural landscape and the slight structural variation allows for the capture of some energetic minima into the crystal structures which are otherwise inaccessible in a structurally similar system.

Having demonstrated the robustness of the synthon approach through retrieving alternative structural polymorphs, we now show how the synthon approach can be further used for relatively complex systems involving co-crystals. Co-crystals are multicomponent crystals sustained by robust and reliable *heterosynthons*. Co-crystallization in itself is a challenging task as it involves recognition between two different molecules and thus co-crystals are formed only when the formation of *heterosynthons* is preferred over that of *homosynthons*.^42^ In other words, the formation of co-crystals is favored only when the resulting structure is lower in energy compared to the native structures of the individual components. Although it is often difficult to predict the formation of co-crystals, they offer additional flexibility in terms of the design providing a handle on their properties. Recently, pharmaceutical co-crystals have received significant attention in view of their improved physicochemical and pharmacokinetic properties.^42,43^ Analogously, 2D co-crystals consist of crystalline supramolecular networks made up of two or more building blocks. They offer additional flexibility in terms of design and control and therefore can be tuned rationally when applying the principles of (2D) crystal engineering. The fabrication of porous co-crystal nanostructures only adds to the persisting difficulty of co-crystallization given the inherently poor stability of low-density networks. We illustrate below that minor structural modifications of the parent compound can change the balance of interactions in such a way that 2D co-crystallization with a given modified molecule (**2**) proceeds much more readily relative to that involving the parent compound (**1**, *vide infra*) itself.

Given the challenges associated with the 2D co-crystallization of porous networks at the solution–solid interface, early attempts to make 2D co-crystals involved the use of stronger and more directional interactions such as hydrogen bonding between carboxyl groups,^44^ and acid–pyridine interactions.^45^ The use of weak directional interactions such as halogen bonds in the fabrication of co-crystals is still in its infancy. Halogen bonding has emerged as an important non-covalent interaction in the recent past and is highly sought after for the fabrication of functional materials.^46^ In verbatim, a halogen bond, R–X···Y–Z, is said to have formed when there is evidence of a net attractive interaction between the electrophilic region of a halogen atom X belonging to a molecule or a molecular fragment, R–X (where R can be another atom, including X or a group of atoms) and the nucleophilic region of a molecule or a molecular fragment, Y–Z.^46,47^ Although halogen bonds are similar to hydrogen bonds in terms of the nature of the interaction, they differ from hydrogen bonds in terms of strength and directionality.^48^ Halogen bonds are moderately strong and occupy a middle ground between strong and weak hydrogen bonds. In contrast to hydrogen bonding, halogen-bonding is considered hydrophobic. Last but not the least, the strength of halogen bonding can be readily tuned through the choice of halogen and often heavier organic halogens lead to relatively stronger halogen bonds. Halogen bonding has enormous potential in designing crystal structures and is being considered as an additional tool along with hydrogen bonds to diversify the structure and function of co-crystal networks.^48–50^

It was recently shown that compound **1** can be 2D co-crystallized together with strong halogen bond donors such as **4F2I** and **3F3I** (Fig. 1) on the HOPG surface.^34^ This strategy benefited from the high adsorption affinity of **1** and strong interactions between the halogen bond donors and the tripyridine acceptor. While 2D co-crystallization of **1** with **4F2I** led to the formation of a porous network stabilized by halogen as well as weak hydrogen bonds (such as C–H···F), a purely halogen bond based porous structure was obtained when **1** was co-crystallized with **3F3I** (see Fig. S8 and S9 in the ESI†). These co-crystal networks however, could only be obtained using a special deposition method. The STM tip was pre-loaded with the two components by immersing it in a solution mixture containing the two components. Several voltage pulses with a magnitude of ∼3.6 V were applied to the STM tip while it scanned the HOPG surface in a thin film of 1-phenyloctane. This so-called ‘electric manipulation’ was essential for the fabrication of halogen-bonded co-crystals. The drop-casting of a 1-phenyloctane solution containing the two components only yielded the mono-component close-packed **1_n_P2** structure of **1**.^34^ This clearly indicates that the parent **1_n_P2** structure is relatively more stable compared to the halogen-bonded co-crystal and thus interferes with the formation of the co-crystal. As nanotechnology generally seeks equilibrium based methods to create nanostructures over a large area through self-assembly, it is essential that such hybrid co-crystal networks are formed at or close to equilibrium conditions. We hypothesized that the structural perturbation of the parent building block might affect the co-crystallization behavior of the compound as it prohibits the formation of the parallel dimer synthon **P2** and it would also facilitate the formation of the desired co-crystal network under equilibrium conditions.

**Fig. 5:**
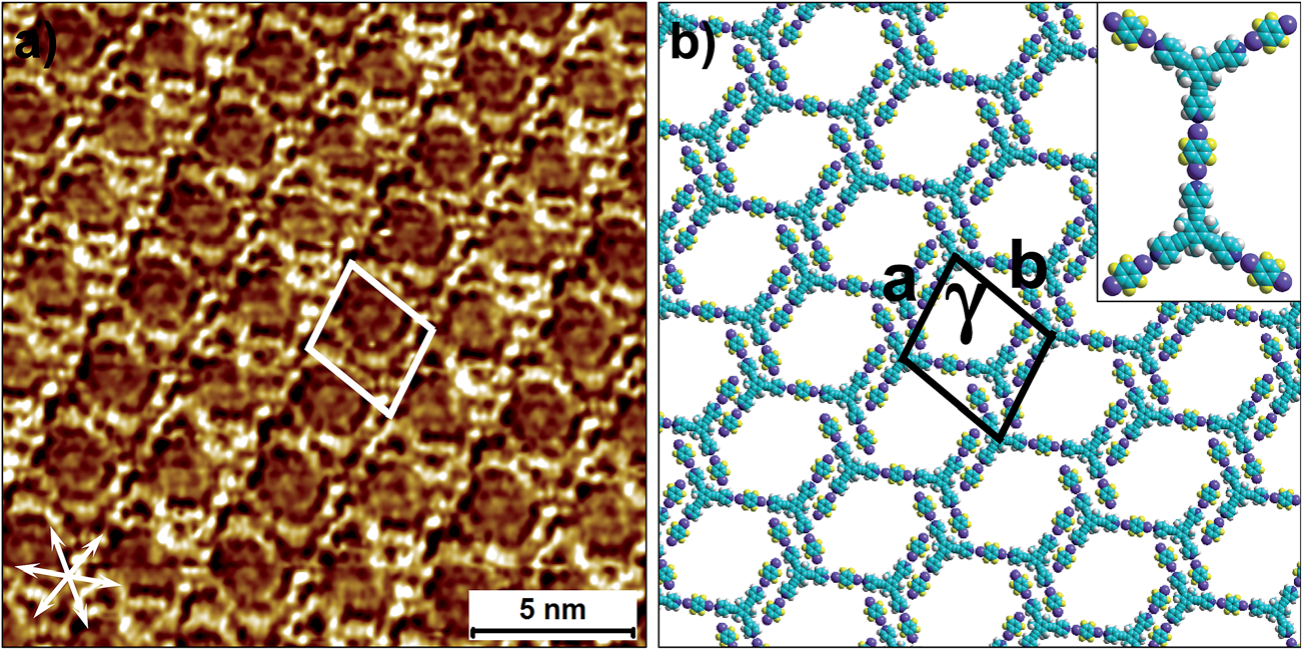
Halogen-bond based 2D co-crystal formed through the co-adsorption of **2** and **4F2I**. (a) STM image showing the porous **2-4F2I** network. Imaging parameters: *V*_bias_ = –500 mV and *I*_set_ = 100 pA. (b) Molecular model depicting the arrangement of the two molecules within the 2D co-crystal. The inset shows the basic halogen-bonded unit. The unit cell parameters are provided in Table 2.

To this end, co-crystallization experiments of **2** with halogen bond donors **4F2I** and **3F3I** were carried out with pre-mixing the two components in 1-phenyloctane. 100 μL each of a 25 μM solution of **2** and saturated solutions of halogenated compounds were mixed together to ensure 2D co-crystal formation. An excess of the small halogenated compounds was used in solution to compensate for their (anticipated) lower adsorption enthalpy relative to **2**. Drop-casting a pre-mixed solution containing **2** and **4F2I** onto the HOPG surface led to the formation of the anticipated 2D co-crystal stabilized by halogen- as well as hydrogen-bonding interactions. Fig. 5 shows the STM image of the **2-4F2I** co-crystal formed at the 1-phenyloctane/HOPG interface (also see Fig. S8 in the ESI†). The 2D co-crystal domains span several thousand square nanometers and the network was found to be stable under STM scanning for several hours. The cavities of this low-density network appear empty and are possibly filled by mobile 1-phenyloctane molecules. The unit cell contains 7 molecules: 2 molecules of **2** and 5 molecules of **4F2I**. The unit cell parameters of the **2-4F2I** co-crystal network are comparable to that of **1-4F2I** already reported.^34^ Given the relatively weak nature of halogen bonding interactions, it is plausible that the **2-4F2I** network is stabilized by other interactions as well. Thus, apart from the primary C–I···N_pyr_ halogen bonding, additional hydrogen bonding interactions involving the fluorine atoms (C–H···F) might also be at play. Furthermore, it is also possible for the iodine atoms to show dual character whereby the nucleophilic region of iodine is involved in a weak C–H···I interaction (see Fig. S8†).

**Fig. 6:**
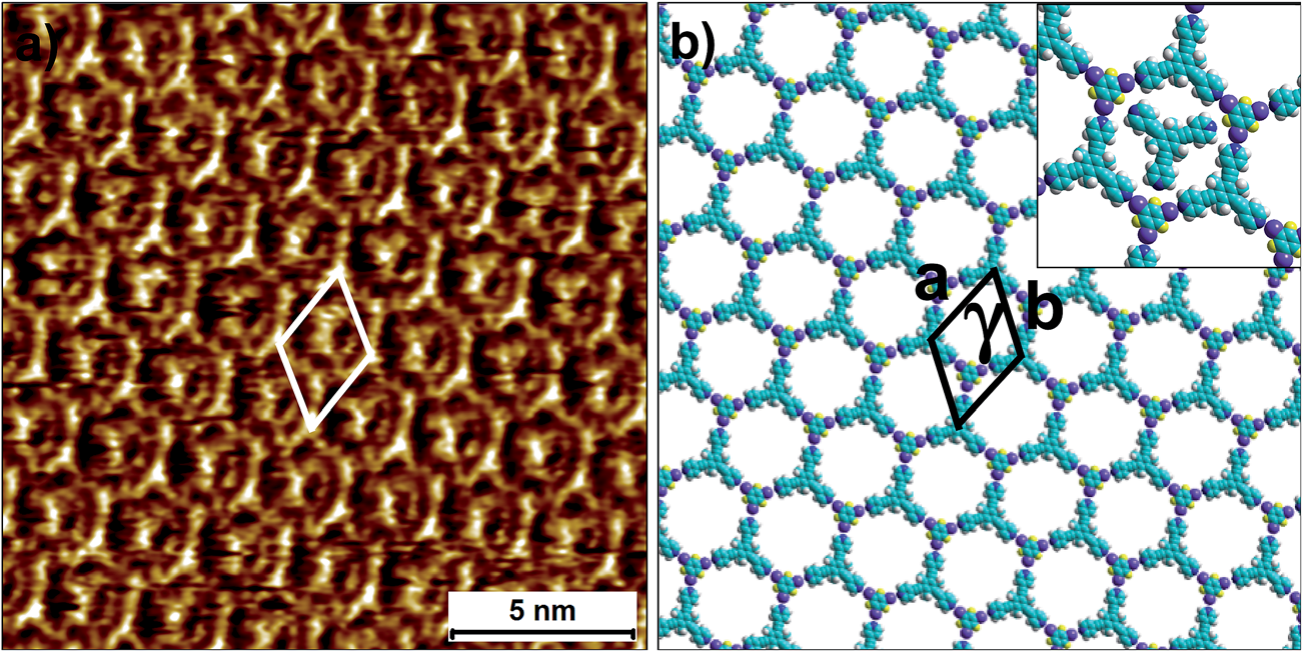
Halogen-bonded 2D co-crystal formed through the co-adsorption of **2** and **3F3I** at the 1-phenyloctane/HOPG interface. (a) STM image showing the porous **2-3F3I** network. Imaging parameters: *V*_bias_ = –1200 V and *I*_set_ = 50 pA. (b) Molecular model depicting the arrangement of the two molecules within the 2D co-crystal. The inset shows the orientation of the trapped guest molecules of compound **2**. The unit cell parameters are provided in Table 2.

The 2D co-crystallization experiments carried out using a combination of compound **2** and **3F3I** revealed an interesting behavior. The deposition of a pre-mixed solution containing the two components onto the HOPG surface yielded the anticipated hexagonal network (Fig. 6). This **2-3F3I** network is sustained by halogen bonds between the pyridine nitrogen of **2** and the iodine atom of **3F3I** (see Fig. S9 in the ESI†). However, the cavities of this network are always occupied with a molecular guest which appears triangular in shape (see the inset in Fig. 6b). We attribute these features to molecules of compound **2** immobilized inside the hexagonal cavities. This assignment is made on the basis of the size measured from the STM data. It must be noted here that **3F3I** has already proven to be an interesting building block in bulk crystal engineering and it has been shown in the past that choosing a guest with the right size directs the structural pattern from linear (such as infinite chains) to a honeycomb structure.^51–53^ A unique feature of this auto host–guest structure is that the orientation of the guest molecules always appears to be the same in all the host cavities whenever the approximately triangular shapes of the guest molecules are resolved in the STM images. The guest molecule, in principle, can adopt two different orientations within the host cavity. However, the STM images show that the guests are always immobilized in the same orientation. A close inspection of the STM images and comparison of the STM data with the molecular model provided in Fig. 6b reveals that the guest molecules adsorb with the pyridinic nitrogens facing the fluorine atoms of **3F3I** (see the inset in Fig. 6b). This structural assignment is somewhat counterintuitive given that nitrogen–fluorine interactions are known to be relatively weak and do not formally qualify as halogen bonds.^48^ However, it has been recently argued based on computational studies that fluorine can participate in non-covalent bonding with electron donors if the acceptor group is sufficiently electron-withdrawing.^54^ In the present case however, simple molecular mechanics based models reveal the N···F distance to be ∼4.4 Å, indicating the absence of attractive N···F interactions. This points towards a rather simple explanation where the steric hindrance of the methyl groups of the host molecules rotationally locks the guest molecules in the observed configuration. The basis of this auto host–guest system appears to be the size complementarity between **2** and the host cavity of the **2-3F3I** network.

Another interesting feature of the **2-3F3I** network is that the network does not exist without the guest species. Reducing the relative concentration of compound **2** in the solution mixture did not yield any self-assembled networks. This observation indicates that the presence of the molecular guests is a necessary condition for the existence of the **2-3F3I** co-crystal network. In contrast to this observation, the previously reported **1-3F3I** network was formed without any guest species. The difference in the two systems possibly arises from the anticipated differences in the adsorption enthalpies of **1** and **2**. As mentioned before, the presence of three methyl groups on the central phenyl ring reduces the effective π–π interactions between **2** and the substrate relative to those in the case of **1**. It is also plausible that the guest molecules are incorporated into the network during the assembly of the host network.

Both of the porous co-crystal networks discussed above are capable of hosting molecular guests and thus could be of interest for host–guest chemistry. In the case of the **2-3F3I** system this is already exemplified by the adsorption of molecules of **2** in the host cavities. On the other hand, the addition of coronene to the co-crystal network of **2-4F2I** yielded a host–guest system where the coronene molecules were found to be immobilized inside the cavities of the host network. The unit cell parameters of the **2-4F2I** network did not change upon coronene adsorption, illustrating the robustness of the halogen-bonded co-crystals towards guest adsorption (see Fig. S11 in the ESI†).

The experimental results presented above clearly reveal that substitutional variation is a promising approach for sampling the different packing possibilities within the average landscape of structurally similar compounds. Thus, the packing landscape of all the structures discussed above, namely compound **1**, compound **2** and **BCNB**, can be considered to be similar. The variations observed in the structural patterns merely represent possibilities along the crystallization pathway and a given structural pattern can be accessed *via* a variation in the experimental conditions. The co-crystallization experiments described above reveal that minor structural modification also allows for the fabrication of relatively complex assemblies made up of more than one component. The substitution of the methyl groups on parent compound **1** in the present case allows relatively straightforward 2D co-crystallization with halogen bond acceptors *via* simple dropcasting, without the need for electric manipulation as was required for the parent compound (*vide supra*).^34^

However, it must be noted that 2D crystallization on solid surfaces is governed by subtle intermolecular and interfacial interactions and as a consequence, the decisiveness with which one can predict the outcome of the process is often limited. Nonetheless, this limitation can be eased by employing the supramolecular synthon approach where one identifies the network not based on the building block itself but on the specific structural elements that define the network, as illustrated above. The synthon approach is at the core of the concept of the structural landscape, which helps extract relationships among experimentally observed patterns of different compounds that belong to the same chemical family. Such generalization is a first step towards the deterministic prediction of surface-confined supramolecular networks.

## Conclusions and outlook

Although 2D crystal engineering has enormous potential for bottom-up nanofabrication processes, the outcome of 2D crystallization on solid surfaces is often difficult to predict given the multitude of subtle interactions involved in the process. This is further exacerbated by the lack of a systematic compilation of the structural data of the assembling systems. Most 2D crystallization experiments to date have been investigated in an isolated fashion without a significant focus on the generalization of 2D crystallization principles, at least within chemically identical families of compounds. A first step towards the compilation of a comprehensive database that allows the formulation of such generalizations would involve the identification of structural elements that define the 2D crystallization of molecules that belong to the same chemical family. An important aspect that needs to be kept in mind while moving forward is that the outcome of 2D and 3D (bulk) crystallization for a given system, as has been demonstrated already for a few cases, is often very different. The 2D structure only represents a single-layered slice through the corresponding 3D crystal structure when the systems are designed to behave that way or when the intermolecular interactions are extremely strong thus subduing other subtle interactions that underpin the 2D crystallization process. In order to have a more global view of 2D crystallization, one needs an alternative approach, where instead of comparing the crystallization of a given system in 2D *versus* that in 3D, one borrows and employs the well-defined and well-developed concepts from traditional crystal engineering to enhance the general understanding of 2D crystallization processes.

We have illustrated this new approach by employing the concepts of supramolecular synthons and structural landscapes from traditional crystal engineering to the 2D crystallization of a weakly interacting system. Using the concept of structural landscapes, we illustrated that the experimentally observed structural patterns merely represent a sub-set of an average landscape of multiple 2D crystallization possibilities. Minor structural modifications on the building blocks and/or variations in the experimental conditions allow access to alternative structures. This structural perturbation strategy eases the 2D co-crystallization process with halogen bond donating compounds to yield porous bi-component supramolecular networks in a straightforward fashion under equilibrium conditions. The fabrication of such porous architectures has proven challenging due to the weak nature of the supramolecular interactions involved, as well as the competition between the close packed native structures and the resulting low-density co-crystal structures. We further demonstrate that these halogen-bonded porous networks are robust and can be employed to immobilize molecular guests such as coronene.

The integrated approach presented above, where one can identify the self-assembling systems based on the primary assembling units, bodes well for the rapidly growing field of nanotechnology which often seeks a robust and predictable outcome of 2D crystallization processes. Such a holistic approach explores the mutual relationship between the structural patterns of a given system and attempts to use this insight in structural design. Considering the observed supramolecular structures as part of an average profile will lend insight towards rational molecular engineering which could be successfully implemented in the design and fabrication of complex supramolecular systems. The idea of structural landscapes is new in two dimensional self-assembly but may be extremely useful for tuning supramolecular networks especially when the calibration of subtle and weak interactions is required. The development of design strategies based on weak interactions will diversify the scope of 2D crystallizations and may provide further opportunities to tune the structure and thus the function of physisorbed thin films of organic molecules.
