# Supplementary material for: Two-dimensional crystal engineering using halogen and hydrogen bonds: towards structural landscapes
Source: Chem Sci. 2017 Mar 16;8(5):3759–69. doi: 10.1039/c7sc00129k (PMC5427994; doi:10.1039/c7sc00129k)
Supplement: Supplementary file 1 [file SC-008-C7SC00129K-s001.pdf]

Electronic Supplementary Material (ESI) for Chemical Science.
This journal is © The Royal Society of Chemistry 2017

Supporting Information

Two-Dimensional Crystal Engineering using Halogen and Hydrogen Bonds: Towards Structural Landscapes

Arijit Mukherjee,† Joan Teyssandier,† Gunther Hennrich,‡ Steven De Feyter†* and Kunal S. Mali*†

†Division of Molecular Imaging and Photonics, Department of Chemistry, KU Leuven–University of Leuven, Celestijnenlaan 200F, B3001 Leuven, Belgium, ‡Universidad Autonoma de Madrid, Cantoblanco, 28049 Madrid, Spain

Corresponding authors:

Steven De Feyter (steven.defeyter@kuleuven.be)

Kunal Mali (kunal.mali@kuleuven.be)

Contents:

1. Experimental details – STM.
2. Primary synthons for compound **1**.
3. Schematic showing the arrangement of molecules of compound **2** in the **2_nA1** network
4. Schematic showing the arrangement of molecules of compound **2** in the hypothetical **2_nP2** network
5. Chiral domains of the **2_mA1** network.
6. Molecular model depicting the co-adsorption of solvent molecules within the **2_sA1** network.
7. Additional STM images of the three different supramolecular patterns formed by **2.**
8. Schematic showing the arrangement of molecules in the **2/4F2I** co-crystal.
9. Schematic showing the arrangement of molecules in the **2/3F3I** co-crystal.
10. Additional STM images of the co-crystal networks formed by **2** with halogen bond donors **4F2I** and **3F3I**.
11. Host-guest chemistry inside the halogen bonded co-crystal: **2/4F2I**-Coronene system.

Experimental Section:

STM experiments

All experiments were performed at room temperature (RT, 21－24°C) using a PicoSPM (Keysight Technologies) or Nanoscope IIIa (Bruker) machine operating in constant-current mode. STM tips were prepared by mechanical cutting from Pt/Ir wire (80%/20%, diameter 0.25 mm). Compound **2** was synthesized and purified using a procedure already reported in the literature.[1] 1-phenyloctane (Sigma 98%) was used as the solvent without further purification. Prior to imaging, solids of compounds **2**, **4F2I** (Sigma-Aldrich 98%) and **3F3I** (Acros 97%) were dissolved in 1-phenyloctane in appropriate amount and a droplet of the sample solution (or solution mixture) was applied by a pipette onto a freshly cleaved surface of highly oriented pyrolytic graphite (HOPG, grade ZYB, Advanced Ceramics Inc., Cleveland, USA). A 250 μM solution of **2** was prepared in 1-phenyloctane first and dilution series was prepared further. For co-crystallization experiments, equal volumes (100 μL) of saturated solutions of either **4F2I** or **3F3I** and 25 μM solution of **2** were premixed in a vial. The vial was gently sonicated and a droplet of the premixed solution was applied on HOPG surface. The experiments were repeated in several sessions using different tips to check for reproducibility and to avoid experimental artifacts, if any. Several samples were investigated, and for each sample, several locations were probed. For analysis purposes, recording of a molecular image was followed by imaging the graphite lattice underneath it under the same experimental conditions, except for lowering the bias. The images were corrected for drift *via* Scanning Probe Image Processor (SPIP) software (Image Metrology ApS), using the recorded graphite images for calibration purposes, allowing a more accurate unit-cell determination. The unit-cell parameters were determined by examining at least 5 images and only the average values are reported. After the determination of the unit cell from the acquired STM images, a molecular model of the observed monolayer is constructed using HyperChem™ Professional 7.5 program. First, a molecular model for a single molecule is built, and then this model is duplicated. The model of the entire monolayer is constructed by placing the molecules in accordance with the unit-cell parameters obtained from the analysis of the calibrated STM images Colors used in molecular models for specific elements are as follows. Carbon: Cyan, Hydrogen: White, Nitrogen: Blue, Iodine: Purple, Fluorine: Yellow The STM images provided in the main text are Gaussian filtered. The imaging parameters are indicated in figure captions: sample bias (V_{bias}) and tunneling current (I_{set}).

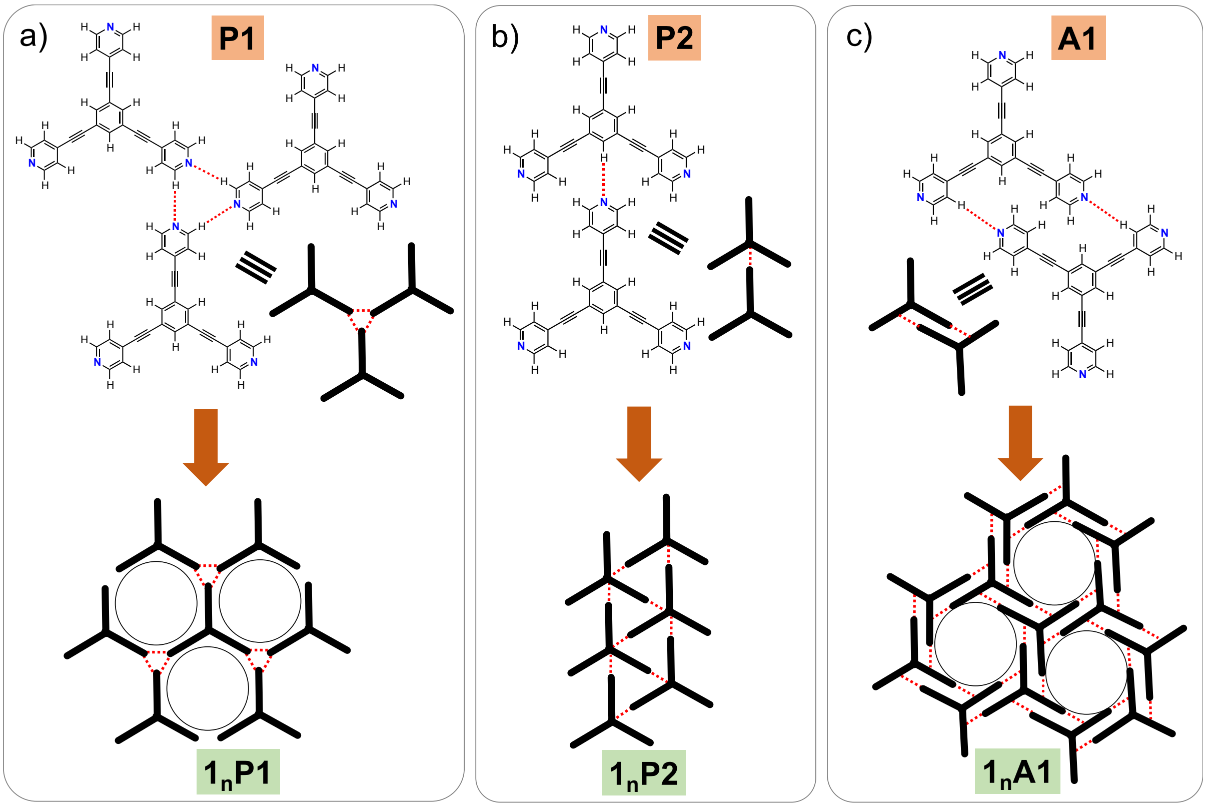

Figure S1. (a-c) Primary synthons based on (aryl)C–H···N(pyridyl) interactions (red dotted lines) for compound **1**. These three primary synthons give rise to three different structures. **P** stands for parallel arrangement of dimers whereas **A** stands for antiparallel arrangement.

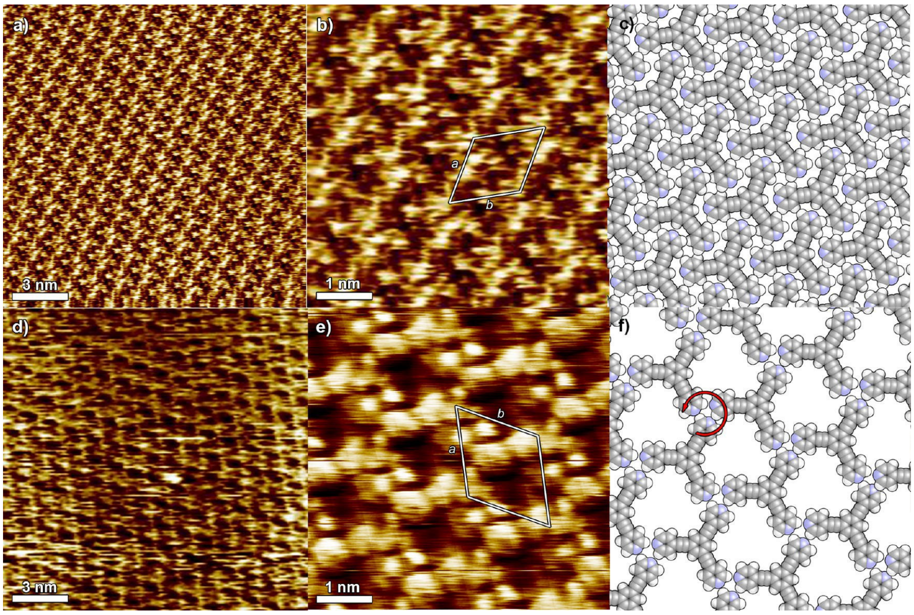

Figure S2. (a-c) STM images and molecular models of the **1_nP2** structure formed by compound **1.** (d-f) STM images and molecular models of the **1_nP1** structure formed by compound **1.** ***<u>(Reproduced from ref. 29 (main text) with permission from the American Chemical Society.)</u>***

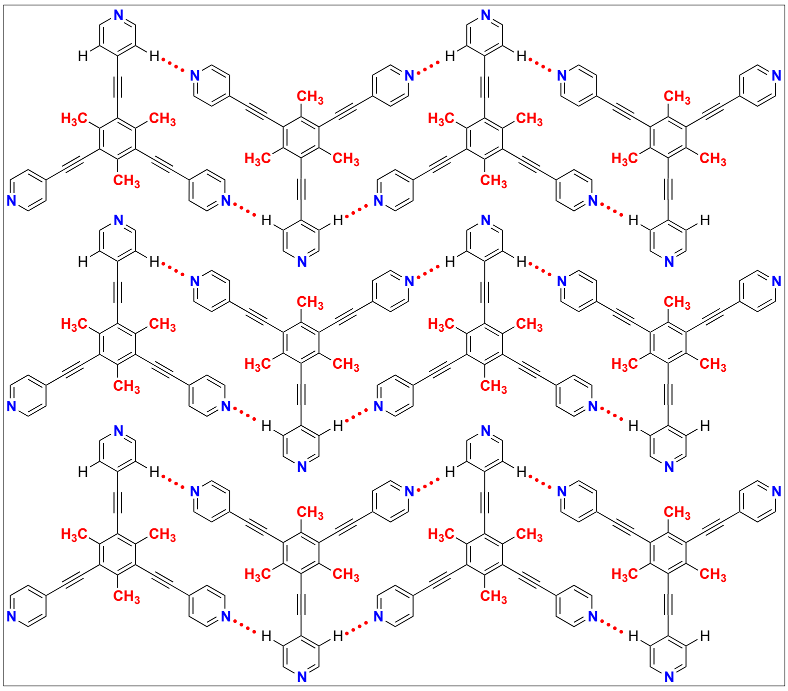

Figure S3. Schematic showing the arrangement of molecules of compound **2** in the $\mathbf{2_nA1}$ network. Each molecule forms four N(pyridyl)---H-C(pyridyl) hydrogen bonds with neighboring molecules. The antiparallel arrangement of two molecules is highlighted in light blue color.

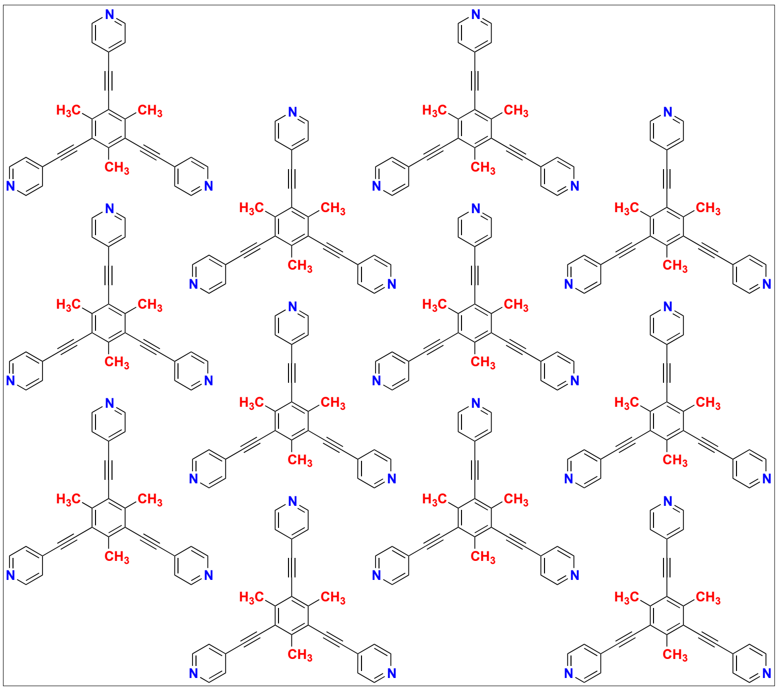

Figure S4. Schematic showing the arrangement of molecules of compound **2** in the hypothetical $\mathbf{2_nP2}$ network. This arrangement of molecules is not observed experimentally. Note that N(pyridyl)---H-C(pyridyl) hydrogen bonds cannot be formed with neighboring molecules when the parallel dimer synthon is adapted. The parallel dimer synthon **P1** is highlighted in blue color.

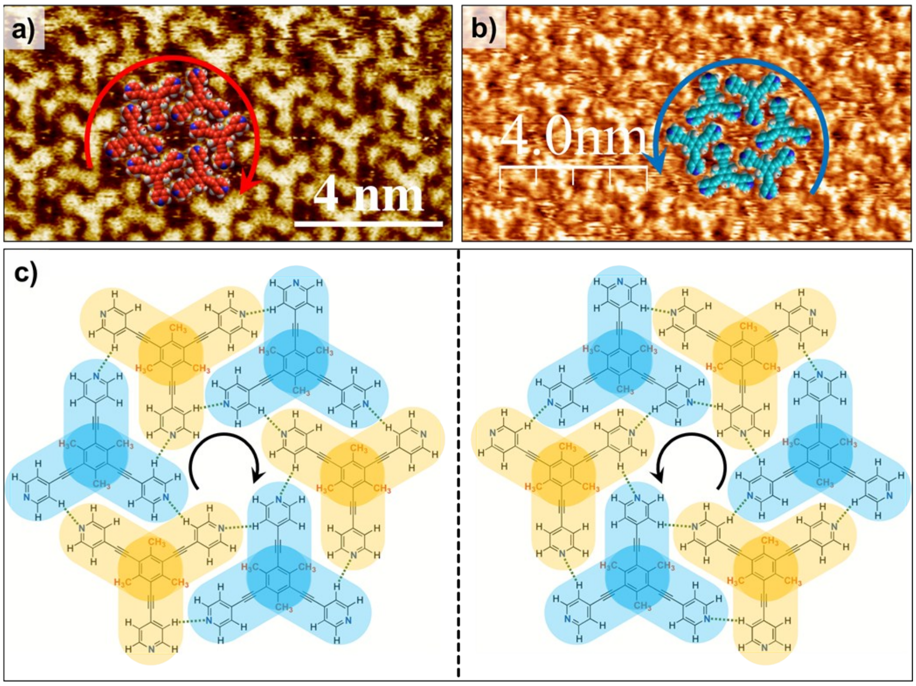

Figure S5. (a, b) STM images showing two types of molecular domains observed in the 2_mA1 network that are related to each other *via* mirror image symmetry. (c) Schematic showing the arrangement of molecules in the opposite handed hexamers. Note that in each case every molecule of **2** forms six N(pyridyl)---H-C(pyridyl) bonds with its neighbors.

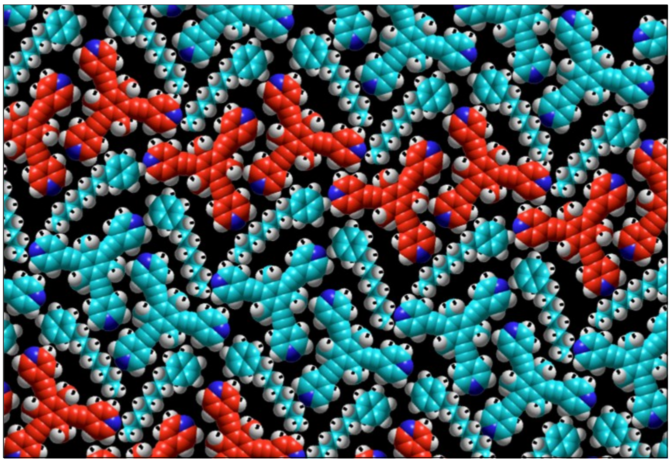

Figure S6. Tentative molecular model showing possible co-adsorption of 1-phenyloctane molecules (the solvent) within the $\mathbf{2_sA1}$ network. The co-adsorption is possibly governed by size and shape complementarity between 1-phenyloctane molecules and the cavities of within the $\mathbf{2_sA1}$ network. Assuming that the 1-phenyloctane molecules adsorb in a planar fashion on the surface (as depicted in the model), the co-adsorption may as well be stabilized by weak C-$H_{phenyloctane}$---N_{Pyr} interactions.

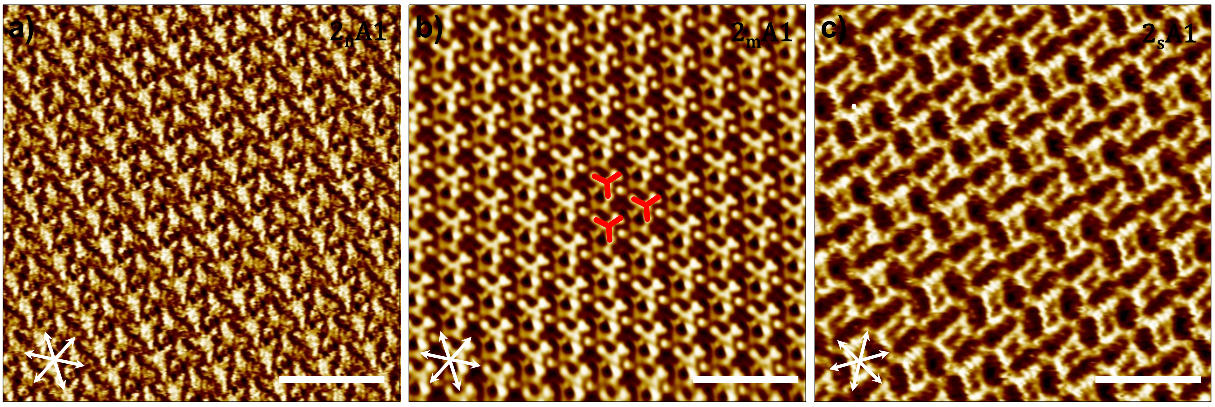

Figure S7. Additional STM images of the three different supramolecular patterns formed by compound **2** at the 1-phenyloctane/HOPG interface. (a) $\mathbf{2_nA1}$, (b) $\mathbf{2_mA1}$ and (c) $\mathbf{2_sA1}$. As mentioned in the main text, $\mathbf{2_nA1}$ and $\mathbf{2_mA1}$ are consistently observed at high (250 μM) and low (25 μM) solutions, respectively. The $\mathbf{2_sA1}$ structure on the other hand was observed rarely upon deposition of dilute (25 μM) solutions. Scale bar for all images = 5 nm. The STM image in panel (b) is Gaussian filtered. It is evident from this as well as the STM image provided in Figure 3c of the main text that, in this packing arrangement three molecules appears brighter than the other three molecules. This contrast variation is possibly a result of the inequivalent registry of the molecules on the HOPG lattice.

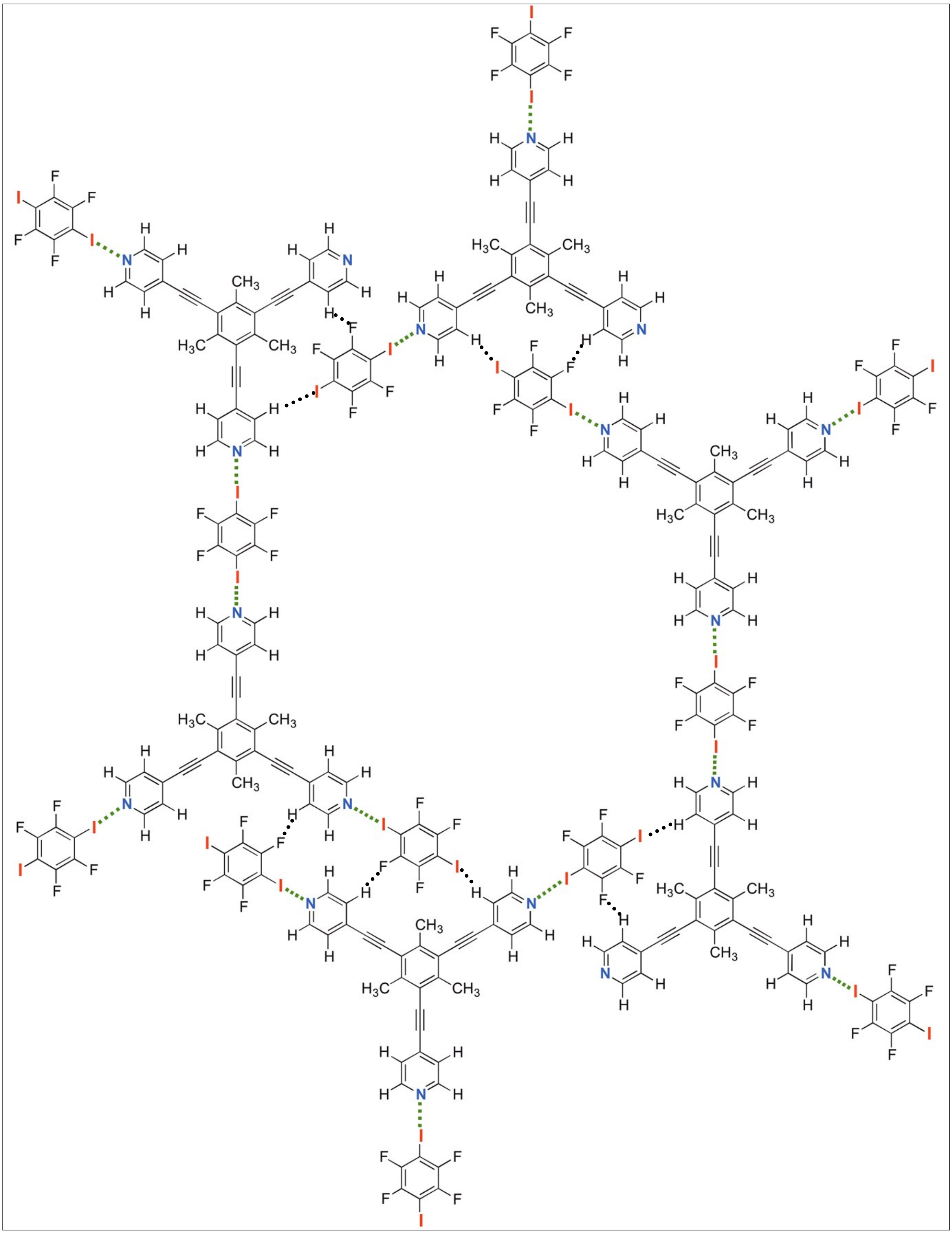

Figure S8. Schematic showing the arrangement of molecules of compound **2** and **4F2I** in the co-crystal network **2/4F2I**. Each molecule of **2** forms three halogen bonds with three molecules of **4F2I**. Additional stabilizing interactions may include hydrogen bonding involving the fluorine atoms (C-H···F) and C-H···I interaction involving the nucleophilic region of iodine. It must be noted that C-H···I interaction is expected to be relatively weak. All interactions are indicated as dotted lines.

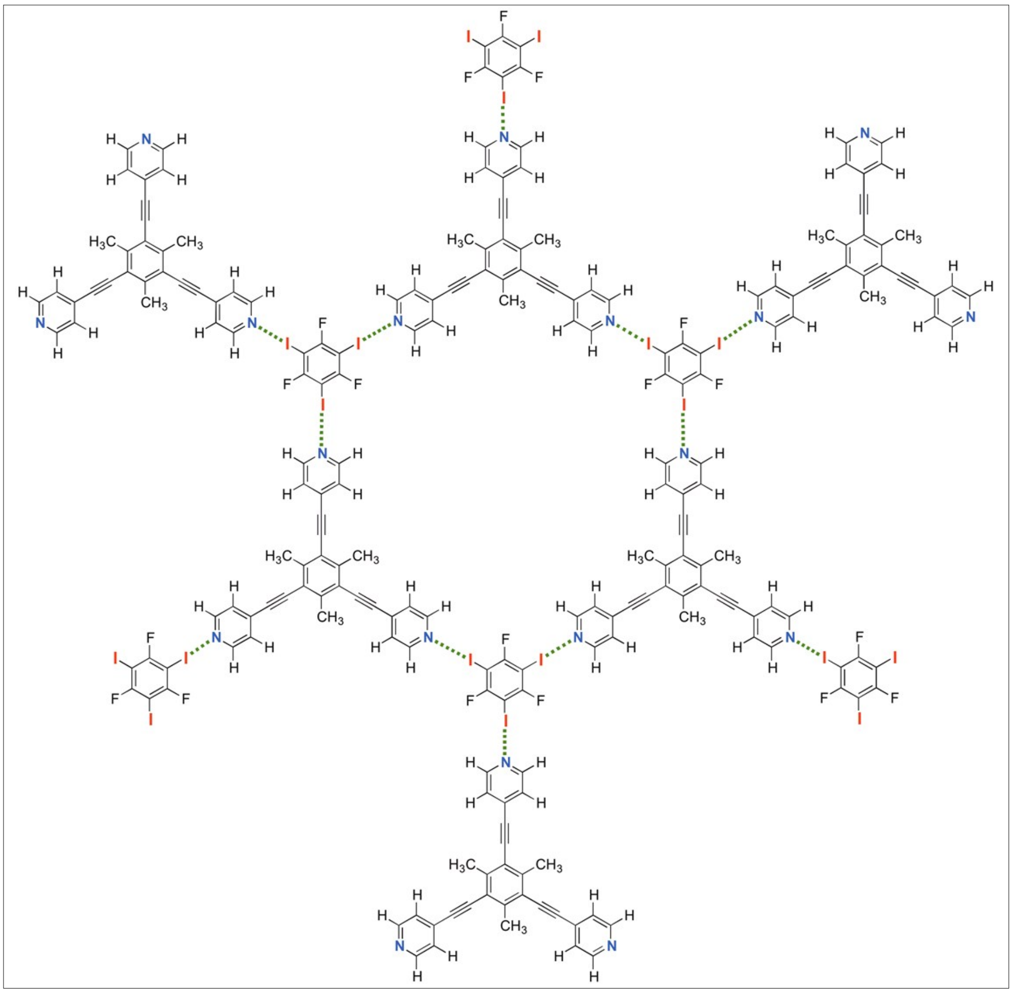

Figure S9. Schematic showing the arrangement of molecules of compound **2** and **3F3I** in the co-crystal network **2/3F3I**. Each molecule of **2** forms three halogen bonds with one molecule of **3F3I**.

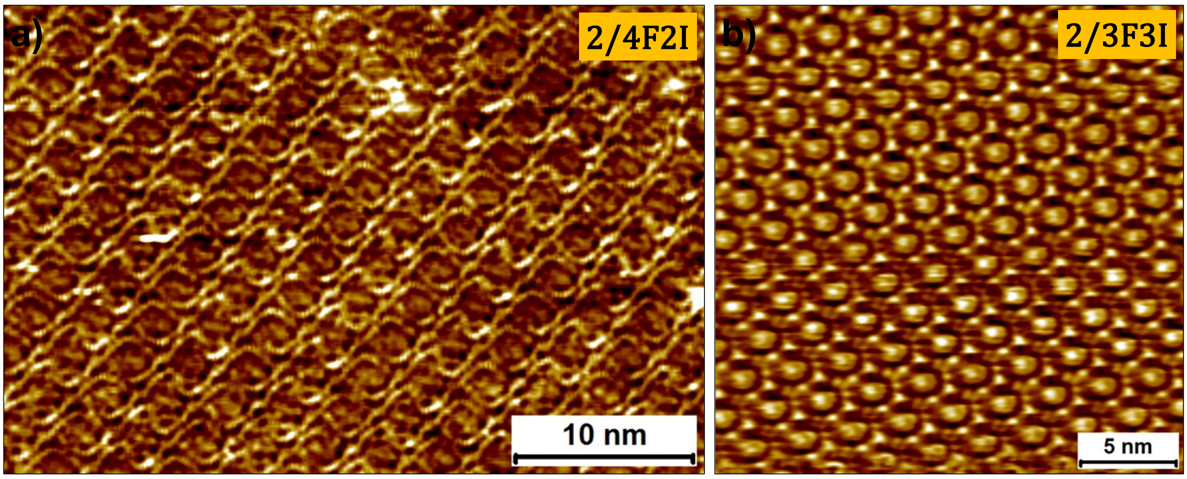

Figure S10. Additional STM images of the co-crystal networks formed by **2** with halogen bond donors **4F2I** and **3F3I**. Note that in contrast to the STM image for the **2/3F3I** system provided in the main text, the image provided in panel (b) does not clearly resolve the molecular guests in the host cavities.

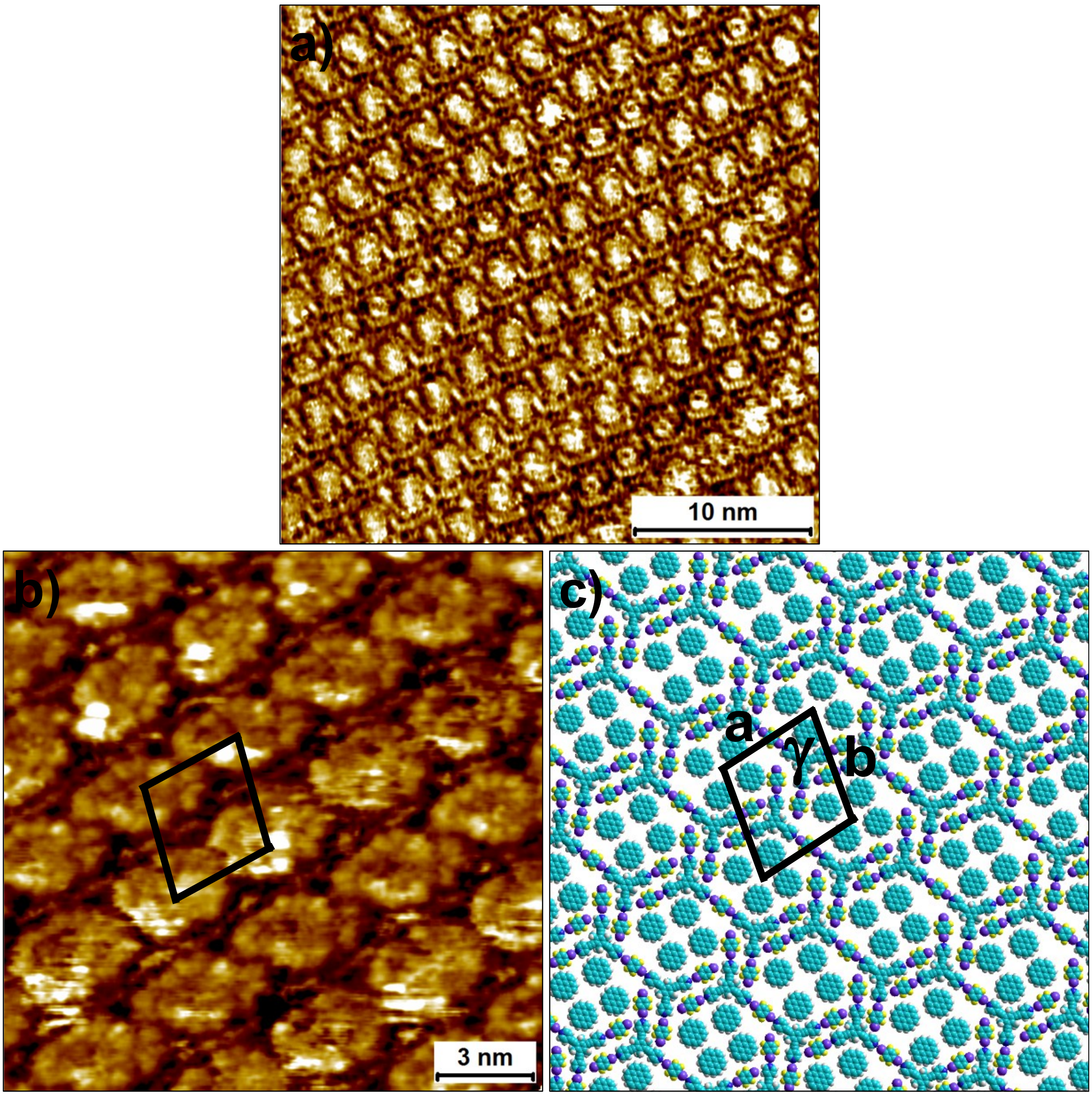

Figure S11. Host-guest chemistry inside the halogen bonded co-crystal. (a, b) STM image showing the immobilization of coronene inside the cavities of the co-crystal network **2/4F2I** at the 1-phenyloctane/HOPG interface. Saturated solution of coronene was added to the pre-formed network of **2/4F2I**. (b) Proposed molecular model for the **2/4F2I-coronene** host-guest system. Although coronene molecules are not clearly resolved, the model suggests that up to four molecules of coronene can be accommodated inside the cavities.

References:

[1] I. Asselberghs, G. Hennrich and K. Clays, *J. Phys. Chem. A* **2006**, *110*, 6271-6275.
